# Efficacy of stellate ganglion block with an adjuvant ketamine for peripheral vascular disease of the upper limbs

**DOI:** 10.4103/0019-5049.72645

**Published:** 2010

**Authors:** Kalpana R Kulkarni, Anita I Kadam, Ismile J Namazi

**Affiliations:** Department of Anesthesia, D.Y. Patil Medical College, Kolhapur, Maharashta, India

**Keywords:** Ketamine, peripheral vascular disease, stellate ganglion block

## Abstract

Stellate ganglion block (STGB) is commonly indicated in painful conditions like reflex sympathetic dystrophy, malignancies of head and neck, Reynaud’s disease and vascular insufficiency of the upper limbs. The sympathetic blockade helps to relieve pain and ischaemia. Diagnostic STGB is usually performed with local anaesthetics followed by therapeutic blockade with steroids, neurolytic agents or radiofrequency ablation of ganglion. There is increasing popularity and evidence for the use of adjuvants like opioid, clonidine and N Methyl d Aspartate (NMDA) receptor antagonist – ketamine – for the regional and neuroaxial blocks. The action of ketamine with sympatholytic block is through blockade of peripherally located NMDA receptors that are the target in the management of neuropathic pain, with the added benefit of counteracting the “wind-up” phenomena of chronic pain. We studied ketamine as an adjuvant to the local anaesthetic for STGB in 20 cases of peripheral vascular disease of upper limbs during the last 5 years at our institution. STGB was given for 2 days with 2 ml of 2% lignocaine + 8 ml of 0.25% bupivacaine, followed by block with the addition of 0.5 mg/kg of ketamine for three consecutive days. There was significant pain relief of longer duration with significant rise in hand temperature. We also observed complete healing of the gangrenous fingers in 17/19 patients.

## INTRODUCTION

The sympathetic supply to the head, neck and upper limb is derived from T1-9 segments, and passes through the stellate ganglion (C5-T1). The stellate ganglion is 2.5 cm×1 cm×0.5 cm and lies over the neck of the 1^st^ rib, between C7 and T1. The most common approach for stellate ganglion block (STGB) is paratracheal, at the level of C6^th^ Chassaignac’s tubercle.[1,2] In 1930, efficacy of STGB was well established by White in USA and Leriche in Europe. In 1933, Labat and Greene reported that injection of 33.3% alcohol can produce satisfactory analgesia. In 1936, Putnam and Hompton first used phenol for neurolysis.[3]

Besides chronic regional pain syndrome (CRPS), sympathetic blockade is found to be useful in circulatory problems of the upper limbs, such as arterial embolism, accidental intra-arterial injection of drugs and Meniere’s syndrome. It has been indicated as immediate therapy for pulmonary embolism.[4,5] Repeated injections of STGB has become popular for the long-term remissions that it produces in CRPS. Serial blocks disorganize the reflex activity triggered in the internunceal neuronal pool of the spinal cord and in the sympathetics themselves.[6] The sympathetic blockade produces relaxation of the upper extremity arteries, which increases blood flow and peripheral temperature.[7,8] Peripheral vascular disease (PVD) of the upper limbs may be due to generalized atherosclerosis, thromboembolism, Buerger’s disease, diabetic angiopathy or Reynaud’s disease. Gradual ischaemia of nerves and tissues activates the sympathetic system, leading to the vicious cycle of pain-vasospasm-ischaemia-gangrene. Treatment is multimodal, with initial trials of alpha blockers, calcium antagonists, pentoxifylline or platelet inhibitors, especially when the obstruction results from spasm. Following failure of the primary line of treatment, patients are usually referred for sympathetic blocks with radiofrequency (RF) electrical thermocoagulation /chemical neurolysis of the ganglion.

Due to non-availability/affordability of RF ablation and to avoid the potential complications of chemical neurolysis, we decided to study the efficacy of ketamine as an adjuvant in enhancing the effects of STGB. Ketamine is known to manipulate the NMDA receptors that trigger the aberrant brain activity in neuropathic pain and control the autonomic dysregulation.[9] Besides good analgesia, it has got a local anaesthetic effect by blocking the Na-channel.[10] At low doses (0.1–0.5 mg/kg) of ketamine, psychotrophic effects are less, and can be managed with benzodiazepines.[11] Considering the fact that opioids have a limited role in established neuropathic pain and for its potential complications,[12] we felt ketamine to be a rational adjuvant for STGB. This report presents the result of 20 cases of PVD of upper limbs with gangrene of fingers, treated by serial STGB for 5 days with local anaesthetic (LA) and ketamine.

## METHODS

A prospective analytical study was performed in 20 patients of PVD of upper limbs during the last 5 years. Approval by the institutional ethical committee and informed consent were obtained. The chief complaints were severe throbbing pain with cold fingers and dry gangrene of 8 days to 1 month duration. Patients were thoroughly assessed and investigated. Laser Doppler flowmetry of the affected extremity was carried out. Patients with h/o recent myocardial infarction /heart blocks and with International normalized ratio (INR) >1.5 were excluded. Pre-block vital parameters, temperature of the normal and affected hand and visual analogue scale (VAS) score for pain were recorded. Diagnostic STGB was carried out with local anaesthetic for 2 days. Patients having pain relief of 50% in the initial magnitude and increase in temperature of the affected hand by 1.5°C were subjected to therapeutic sympatholysis with ketamine. Pre-medication with intravenous midazolam 0.5 mg/kg was given for anxiolysis.

### Technique

**Figure 1:**
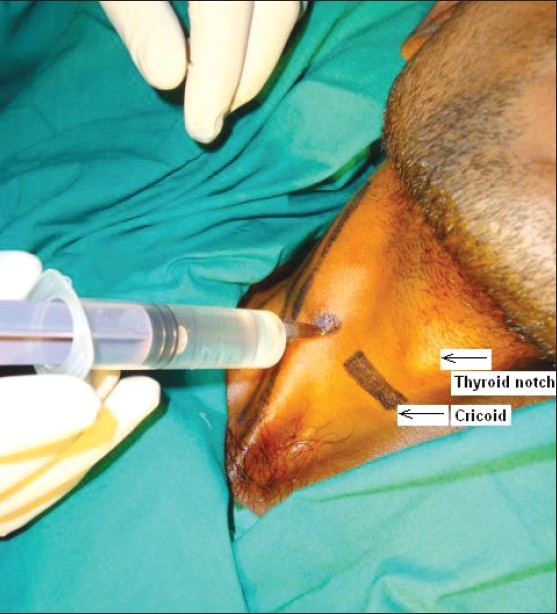
Technique of stellate ganglion block

For classical anterior paratracheal approach at C-6^th^ level, patient was lying supine with extension of head at the A-O joint and mouth partially open so as to relax the muscles in the neck [Figure 1]. After skin preparation, the chassaignac’s tubercle was palpated at the level of the cricoid cartilage (1.5 cm from midline) and the sternocleidomastoid muscle and the carotid vessels were retracted with one hand. Under C-arm guidance, a 21 G 5-cm needle was inserted perpendicular to the table to hit the C-6^th^ tubercle approximately at a depth of 2–2.5 cm. The needle withdrawn for 2 mm to prevent periosteal or injection into the longus colli muscles. After −ve aspiration test, lignocaine 2% 2 ml+8 ml of 0.25% bupivacaine was injected in increments. Monitoring of vital parameters during the block, after the block for 30 min and, later, hourly for 4 h was carried out in the recovery room. The block was repeated in a similar way on the 2^nd^ day to confirm the benefits. Later, for three successive days, STGB was repeated with 1.5 ml of 2% lignocaine +8 ml of 0.25% bupivacaine +0.5 mg/kg of ketamine. Intramuscular Diclofenac 2 mg/kg or Tramadol 2 mg/kg was given as soon as VAS reached >6.

### Clinical observations

1. For pain relief – (0–10 point) VAS 2-hourly for 8 h and later 4-hourly up to16 h and at 24 h.
2. Baseline axillary temperature and temperature of the affected hand before and after block, then 2-hourly for 8 h, later 4-hourly up to16 h and at 24 h.
3. S/O improved circulation – vasodilatation, skin texture and colour.
4. Horner’s syndrome.
5. Immediate complications like hoarseness, weakness in the limb, respiratory insufficiency, etc.
6. Weekly follow-up for 1 month, then monthly for 6 months and later 6-monthly for pain relief, sense of warmth, appearance of line of demarcation, healing of gangrenous lesion and return of peripheral pulsations.
7. For any delayed complications.

Statitistical analysis of the data is performed using data analysis Tool Park in Microsoft Excel. As the sample size of the study group is 20, analysis of the quantitative data is carried out using Student’s unpaired “*t*”-test for unequal variance, for comparison of mean VAS score, temperature, duration of analgesia, pulse rate and mean blood pressure. Differences are considered as statistically significant if probability (*P*-value) is <0.05 and as highly significant if *P*<0.001, whereas probability of >0.05 is considered as insignificant. Level of significance is 0.001, 0.01 and 0.05, as appropriate. Power of the test is almost 1 whereever it is significant.

## RESULTS

STGB was performed on 20 patients (M/F=19/1) of age 25–65 years, weighing 40–70 kg. The affecting pathologies are mentioned in Table 1.

**Table 1:** Pre-STGB observations

Pathology	Doppler study	No. of patients
Atherosclerotic gangrene	Weak pulsations brachial/radial	8
Thromboembolic gangrene	Absent flow palmar/digital	7
Diabetic dry gangrene	Weak palmer flow	2
Reynaud’s disease with gangrene	Weak radial flow	1 female
Post-traumatic gangrene	Absent digital flow	1
CRPS with oedema	Weak palmer flow	1
Total		20

STGB: Stellate ganglion block, CRPS: Chronic regional pain syndrome

**Figure 2:**
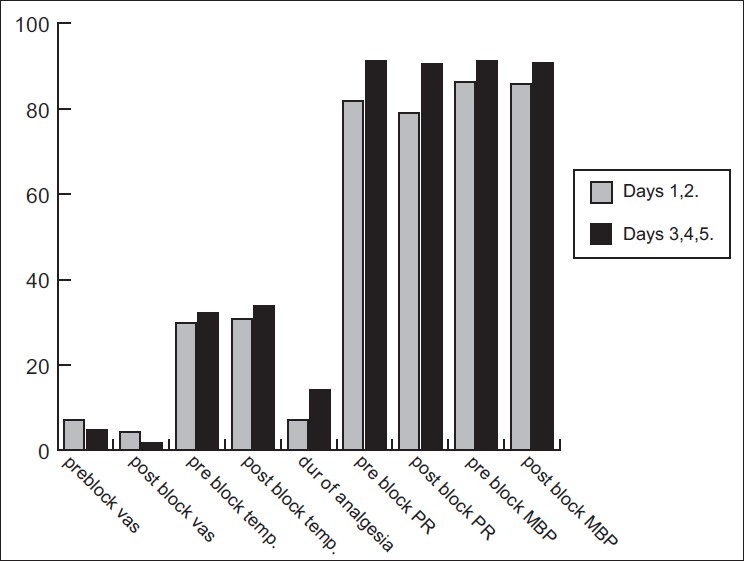
Comparisons of the mean pre- and post-block VAS, temp. and duration of analgesia (h). Comparisons of the mean pre- and post-block PR/min and MBP on days 1 and 2 and days 3, 4 and 5 of STGB

Figure 2 depicts the mean pre-block values of days 1 and 2, where the mean VAS was 7 and the mean surface temperature of the hand was 29.89°C. Following STGB with LA, the mean Post-block VAS was 4.25, with a significant rise in the mean temperature of the affected hand by 0.77°C. (*P*<0.001). The mean duration of analgesia observed was 7 h. Later, the STGB was performed for three successive days with LA+ketamine 0.5 mg/kg. The pre-block mean VAS of days 3, 4 and 5 was 4.7 whereas the post-block mean VAS was 1.6, which was significantly less. The temperature rise obtained was mean 1.73°C. Duration of analgesia was significantly more after the addition of ketamine, with a mean of 14 h (*P*<0.001). There was no significant variation in the mean pulse rate (PR) and mean blood pressure (MBP) before and after block following STGB and with the addition of ketamine.

**Figure 3:**
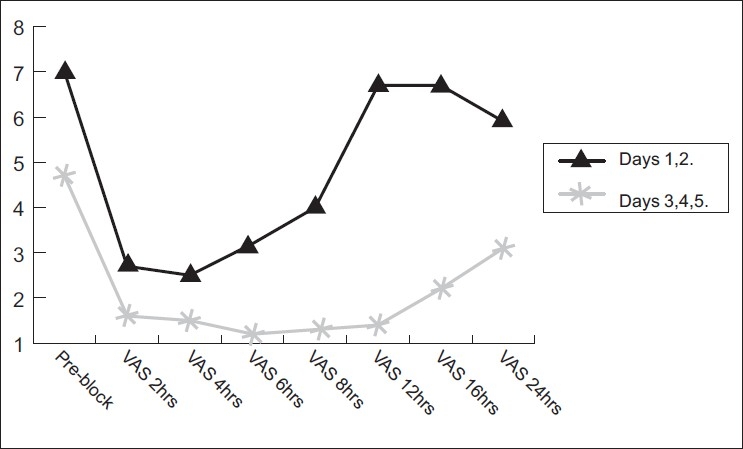
Comparision of the mean VAS after STGB with LA vs addition with ketamine at 2, 6, 12, 16 and 24 h

Figure 3 shows the comparison of the mean VAS of days 1 and 2 (STGB with LA) vs the mean VAS of days 3, 4 and 5 (STGB with adjuvant ketamine). It refers to the VAS record pre and post-block at 2, 6, 12, 16 and 24 h. The pre-block mean (SD) VAS of days 1 and 2 was 7 (0.96); following STGB, it was 2.67 (0.43) and 3.2 (0.6) at 2 and 6 h, which was highly significant (*P*<0.001). At 12 and 16 h, there was significant rise in VAS as 6.7 (0.9) and 6.8 (1.2), respectively, following LA block (*P*>0.05). However, the pre-block VAS of days 3, 4 and 5 achieved was mean (SD) 4.67 (0.7). There was a significant drop in the VAS record as 1.62 (0.73), 1.23 (0.68), 1.39 (0.5) and 2.22 (0.78) at 2, 6, 12 and 16 h, respectively, and it was highly significant (*P*<0.001).

**Figure 4:**
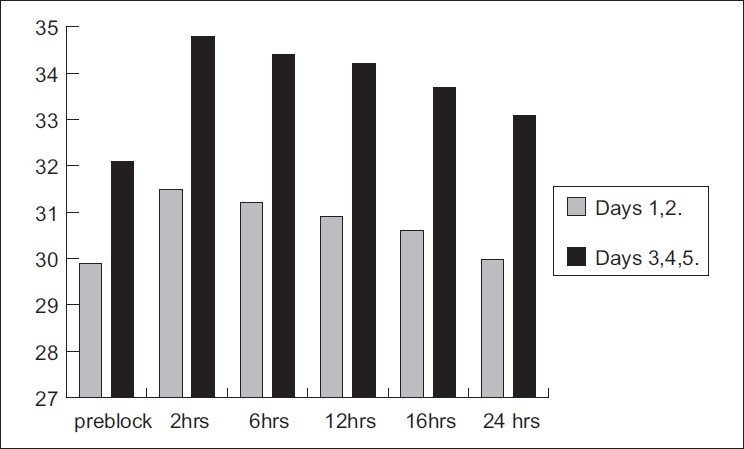
Mean temperature changes in °C in mean of days 1 and 2 and days 3, 4 and 5 at 2, 6, 12, 16 and 24 h

Figure 4 shows the mean post-block temperature rise following STGB with LA on days 1 and 2 and after addition of ketamine on days 3, 4 and 5. It refers to the pre-block mean (SD) temperature of the hand of days 1 and 2, which was 29.89 (1.15)°C. The rise was highly significant at 2, 6 and 12 h, with a significant rise maintained at 16 h following LA block. On days 3, 4 and 5, the pre-block mean temperature achieved was 32.1 (1.23)°C. The mean rise in temperature observed was highly significant at 2, 6, 12 and 16 h following addition of ketamine for STGB.

Table 2 shows the follow-up record at different times for pain relief, warmth and healing of the gangrenous fingers. Hundred percent pain relief was present at the 12^th^ week in (18/20) 94.7% of the patients. Later, few patients were lost to follow-up (LF) from 6 to 24 months. Warmth in the hand was maintained in (19/20) 95% of the cases. Greater than 90% healing of the fingers was observed in (18/19) 94.7% of the patients at the 12^th^ week. The STGB was repeated in two cases, but a diabetic case required amputation of the 2 phalynx at 8^th^ week due to poor response. Repeat Doppler flow study was not possible in all the cases. At 6 months, 17 patients underwent laser Doppler flowmetry, showing improvement in flow from 50% to 90%. At 24 months, 10 patients had 50–100% improvement in the circulation of the hand.

**Table 2:** Follow-up record of % of pain relief, warmth and healing in number of patients, amputation done, no. of lost to follow-up (LF) cases and % of success

Observation (*n*=20)	1^st^ week	2^nd^ week	3^rd^ week	4^th^ week	8^th^ week	12^th^ week	At 6 months	At 12 months	At 24 months	Success at 12^th^ week
Pain relief (no.of patients)	75% (14)	80% (18)	90% (17)	100% (15)	100% (17)	100% (18)	17 (3 LF)	14 (6 LF)	12 (8 LF)	94.7%
Warmth > + (no. of patients)	16	17	17	16	17	19	17	14	12	95%
Healing (no. of patients)	25% (4)	25−50% (9)	50−75% (12)	50−75% (15)	>75% (16)	>90% (18)	100% (17)	100% (14)	100% (12)	94.7%
Repeat block	−	−	−	2						
Amputation				−	1 case	−				5.3%
Doppler study >50% flow							17	12	10	

Table 3 shows that there was transient Horner’s in 12 patients and hoarseness in six patients, which did not require any intervention. Haematoma formation occurred in one case, which subsided after 12 h. Bradycardia occurred in two patients, which responded to 0.2 mg of injection glycopyrrolate. Sixteen patients complained of light-headedness for 10–30 min, which was tolerable following midazolam pre-medication.

**Table 3:** Post-STGB complications in 20 patients

Complications	No. of patients	%
Horner’s	12	60
Hoarseness	6	30
Haematoma	1	5
Dyspnea	0	0
Accidental spinal	0	0
Bradycardia	2	10
Hypotension	0	0
Light headedness	16	80
Psychotropic reactions	0	0
Nausea/vomiting	0	0
Death	0	0

STGB: Stellate ganglion block

## DISCUSSION

STGB is a well-accepted therapeutic technique. Wolf *et al*. observed incidence of minor and short-lived complications in 1.7/1,000 patients, with no major complications in the series of 45,000 cases.[13] The local anaesthetic blocks provide immediate relief but they are not long lasting. Permanent relief may be obtained with repeated blocks using depo-steroids (5–7 times) or with neurolytic agents. The local anaesthetic interrupts the pain–spasm cycle whereas corticosteroids cause membrane stabilization and inhibition of the synthesis/release of pro-inflammatory substances.[14]

Racz *et al*. advocated the injection of 3% phenol (2.5 ml of 6% phenol, 2.5 ml of 0.5% bupivacaine and 80 mg of methylprednisolone) at the stellate ganglion level via the C7 approach under fluoroscopy. They have not observed any long-term Horner’s syndrome.[15] Harris *et al*. in 2006 reported profound pain relief following local application with buprenorphine at the stellate ganglion for head and face pain, supporting the presence of endogenous opioids on sympathetic ganglion.[16] For long-term effects unlike neurolysis of the lumbar sympathetic chain, many clinicians have avoided neurolysis of the stellate ganglion for the risk of producing permanent Horner’s or complications due to spread of neurolytic solution.[1] For prolonged effects, addition of 40 mg of triamcinalone or fentanyl has been advocated.[5,17] Alternatively, RF denervation has been performed at the stellate ganglion to diminish the chance of a permanent Horner’s syndrome.[1,5] However, post-lesioning, neuritis/neuralgias are observed in 10% of the cases and permanent Horner’s or motor paralysis is also reported.[3,17]

Many opioid and non-opioid drugs are tried by different routes to treat chronic pain syndromes.[18] Among them, a NMDA receptor antagonist – ketamine – has gained popularity for variety of its action by multiple routes. In CRPS, the peripheral sensitization of pain with persistent inputs to the dorsal horn of the spinal cord from the C fibres causes the “Wind Up” phenomena.[19,20] The change is either decrease in the inhibitory receptors like GABA or increase in the excitatory receptors like NMDA.[21]

For neuropathic pain, ketamine is reported to be helpful in a dose range of 0.1–7 mg/kg and by infusion for 30 min to 8 h in both CRPS Type I/II patients.[22] Long-term pain relief is observed in CRPS following low-dose infusion for few days with minimal haemodynamic or psychomimetic side-effects.[23]

PVD of the upper limbs present with either acute intractable or chronic ischaemic pain, with discolouration and gangrene of the fingers. To achieve prolonged sympathetic blockade for pain relief and to maintain circulation for healing, ketamine appears to be a good adjuvant for STGB. Its action may be supraspinal by systemic absorption and peripheral through NMDA receptors located on the somatic nerve and dorsal root ganglion. Blockade of peripherally located NMDA receptors is a potential target in the management of neuropathic pain due to vascular insufficiency as well.[24] As there are changes in Na^+^ and Ca^2+^ channels in neuropathic pain, a combination of a Na^+^ channel blocker (local anaesthetic) and NMDA receptor antagonists appears to be rational for the treatment of neuropathic pain. The effect of opioid is decreased in patients with neuropathic pain.[25] Ketamine, on the other hand, is effective after the onset of hyperalgesic symptoms.[21]

The aim of our study was to evaluate the effects of ketamine with STGB. For the first 2 days, STGB was given with 10 ml of local anaesthetic. The mean pre-block VAS score was 7 and post-block score attained at 6 h was 3.2, which was significantly less. Following block with the LA agents, duration of analgesia (time from STGB to first request of analgesics at VAS >6) noted was 7 h (mean of days 1 and 2). After the first record of duration of analgesia, patients received intramuscular Diclofenac 2 mg/kg or Tramadol 2 mg/kg as soon as VAS reached >6. For the first 2 days, intensity of pain was higher in spite of parenteral analgesics. Later, for three successive days, STGBs with the addition of 0.5 mg/kg of ketamine resulted in a pre-block mean VAS of days 3, 4 and 5 of 4.7, showing better response to the parenteral analgesics, and the post-block VAS attained was 1.2 with significantly prolonged duration of analgesia of mean 14 h. The results were similar to the observations noted by Rani sunder *et al*.,[24] who observed a fall in VAS from 10 to 2.5 with STGB with bupivacaine and VAS <1 following addition of 0.5 mg/kg of ketamine for STGB, with duration of analgesia of 36 h in two cases of CRPS. One of our case of CRPS had dramatic reduction in the oedema of the hand within 24 h after single ketamine block. The temperature rise observed following STGB with LA was 1.6°C. and of 2.7°C. with the addition of ketamine. Temperature rise of >1.5°C. was observed following successful sympatheticolysis.[26–28] There are clinical reports of pre-emptive STGB to increase the patency of radial artery grafts in coronary artery bypass surgery[29] and as an indication for the treatment of refractory angina.[30] There are few studies to demonstrate the effect of ketamine on microcirculation when added to sympathetic blockade with LA agents. Probably because of prolonged analgesia due to NMDA antagonism and enhancement of the LA effect by action on Na channels, ketamine helps to maintain the vasodilatation/flow induced by serial STGB. This has resulted in 90% pain relief by the 3^rd^ week in 17 patients and 100% pain relief in 15 patients over 4 weeks duration and also accelerated healing of the gangrenous fingers, as observed in 18/19 patients.

## CONCLUSION

STGB using local anaesthetic with an adjuvant ketamine is a safe and effective technique. Ketamine enhances the effect of sympathetic blockade with relief of pain, ischaemia and maintains circulation. Thus, ketamine is a useful adjuvant to STGB and it obviates the need of permanent destruction of the ganglion by chemical/RF neurolysis in patients with PVD.
